# Sarcopenia in Patients with Osteoporotic Vertebral Fractures is associated with higher risk of Multiple Vertebral Fractures

**DOI:** 10.1007/s11657-025-01633-1

**Published:** 2025-12-04

**Authors:** Biniam Melese Bekele, Ina Moritz, Steffen Reißberg, Yu-Mi Ryang

**Affiliations:** 1 Department of Neurosurgery & Center for Spine Therapy, Schwanebecker Chaussee 50, Helios-Klinikum Berlin-Buch, Berlin, 13125 Germany; 2 Department of Neuroradiology, Helios-Klinikum Berlin-Buch, Berlin, Germany; 3 Department of Neurosurgery, Klinikum Rechts Der Isar, Technical University Munich, Munich, Germany

**Keywords:** Osteoporosis, Sarcopenia, Vertebral fractures, *T*-score, CT Hounsfield units, Bone mineral density

## Abstract

***Summary*:**

Individuals with sarcopenia and osteoporosis often suffer from vertebral fractures. In this study, we show that sarcopenia (reduced muscle mass) is very common in patients with osteoporotic vertebral fractures and triples the risk of suffering from multiple vertebral fractures. Detection of sarcopenia may improve patient care in osteoporosis and prevent future fractures.

**Background:**

Sarcopenia is widely recognized as a significant risk factor for fractures; however, its role in osteoporotic vertebral fractures (OVFs) remains underexplored. This study aimed to evaluate the prevalence of sarcopenia in patients with OVFs and its relationship with clinical characteristics and outcomes.

**Methods:**

This retrospective analysis included 142 patients treated for OVFs at a single institution from January 2022 to June 2024. Quantitative assessments of sarcopenia were performed using axial MRI images at the L4 vertebral level. Measurements included the psoas cross-sectional area (pCSA), which was normalized using the psoas muscle index (PMI = pCSA/height^2^) and the psoas muscle lumbar vertebral body index (PLVI = pCSA/vertebral body area). Fat infiltration (FI) and functional cross-sectional areas (fCSA) were determined using ImageJ®. Sarcopenia was defined using established PMI cutoffs. Clinical parameters, osteoporosis diagnostics (DXA *T*-scores, CT-based Hounsfield units [HU]), and patient outcomes were collected.

**Results:**

Patients had a median age of 81 years (IQR 74–85), and 94 (66.2%) were female. Sarcopenia was identified in 103 patients (72.5%). Patients with sarcopenia had significantly lower BMI, lower serum albumin, and reduced *T*-scores of the lumbar spine compared to those without sarcopenia. Multiple (≥ 2) vertebral fractures were significantly more frequent in patients with sarcopenia (37.2% vs 18%, *p* = 0.018). Logistic regression revealed that patients with sarcopenia were 2.78 times more likely to have multiple fractures (95% CI; 1.1–6.9, *p* = 0.027). Additionally, a significant negative correlation between FI and *T*-scores of the lumbar spine was observed (*r* = −0.242, *p* = 0.037). By contrast, no significant differences were seen in CT HU values, time to postoperative mobilization, length of hospital stay, or incidence of postoperative wound infections.

**Conclusions:**

Sarcopenia is highly prevalent among OVF patients and significantly increases the risk of multiple acute fractures. Assessment and management strategies for OVF patients should routinely incorporate evaluation for sarcopenia.

## Introduction

Osteoporosis is a major contributor to morbidity and mortality in the aging population, with osteoporotic vertebral fractures (OVFs) being the most prevalent type of osteoporotic fractures encountered in clinical practice [1]. In Europe, OVFs have a reported prevalence of 18%–26% and their incidence is projected to rise as the population ages [2]. These fractures not only are a source of significant pain and functional limitation but also are associated with increased mortality and reduced quality of life [3, 4].

Although the association of low bone mineral density (BMD) and increased fracture risk is well established, recent studies have shifted their focus towards identifying additional musculoskeletal risk factors that may influence patient outcomes. Sarcopenia—a progressive and generalized loss of skeletal muscle mass, strength, and function—is increasingly recognized as an important risk factor [5]. As a critical component of the frailty syndrome, sarcopenia is independently associated with a greater risk of falls, fractures, prolonged hospitalization, and mortality among older adults [6].

Furthermore, emerging evidence supports the presence of a pathophysiological interplay in the “muscle-bone unit,” in which muscle deterioration may compromise skeletal integrity [7, 8]. This interaction underpins the concept of osteosarcopenia—the coexistence of osteoporosis and sarcopenia—which may synergistically increase the risk of fracture and adverse outcomes. However, despite the high prevalence of vertebral fractures and the recognized relationship between sarcopenia and decreased bone density, studies specifically investigating the impact of sarcopenia in the context of OVFs remain limited.

The aim of this study was to investigate the prevalence of sarcopenia among patients with OVFs and evaluate its association with clinical parameters and outcomes. Understanding the interplay between muscle and bone health can aid in tailoring interventions and improving the management of these high-risk patient populations.

## Methods 

### Study design

This monocentric, retrospective cohort study was conducted at a single high-volume spine center. Patients treated for OVFs between January 2022 and June 2024 (30 months) were included. Ethical approval was obtained from the local ethics review board (approval number Eth-SB-25-009), and the study complied with the Declaration of Helsinki guidelines.

### Patient selection

Medical records were reviewed for all patients diagnosed with osteoporotic vertebral fractures between January 2022 and June 2024. Patients who fulfilled the following inclusion criteria were included:

1. Age ≥ 18 years.
2. Diagnosis of one or more acute OVFs confirmed in STIR ((Short-Tau-Inversion-Recovery) -weighted magnetic resonance imaging (MRI) and clinical correlation.
3. Availability of axial MRI imaging of the lumbar spine for muscle assessment

**Fig. 1:**
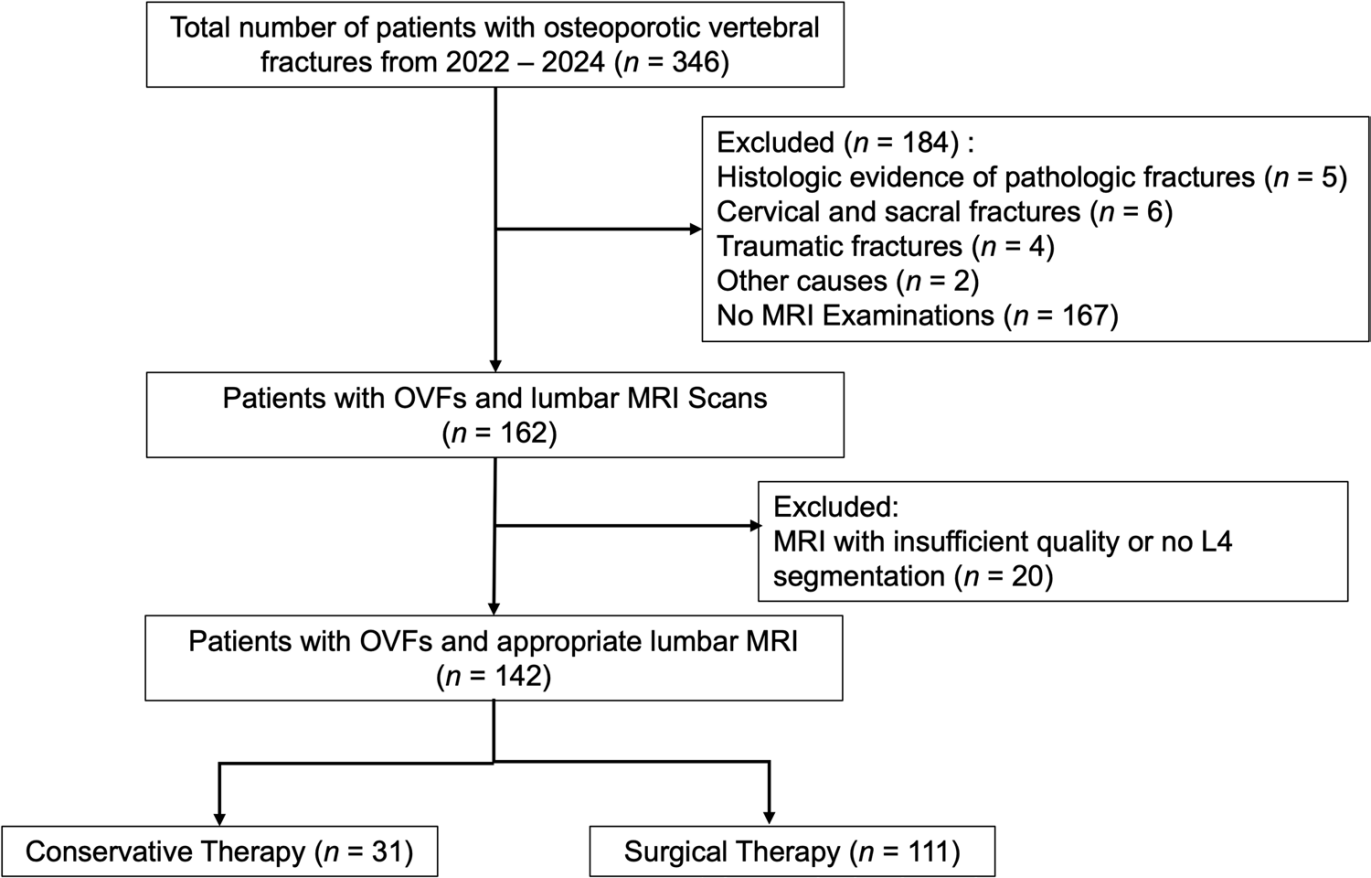
Patient recruitment and selection OVFs – Osteoporotic vertebral fractures, MRI – Magnetic resonance imaging

Patients were excluded if they had non-osteoporotic (traumatic or pathological) fractures, active malignancy, spinal infection, prior lumbar instrumentation or vertebral augmentation of the L4 vertebra, or missing key clinical or imaging data. A total of 346 patients were recruited from medical records, of which 142 fulfilled the inclusion criteria (see Fig. 1).

### Data collection

Demographic data, laboratory parameters, and comorbidities were extracted from patient records. Osteoporosis parameters such as *T*-score were assessed using dual-energy X-ray absorptiometry (DXA). Additional estimation of vertebral bone quality was obtained via computed tomography (CT)-based Hounsfield unit (HU) values measured after a standardized protocol as described earlier [9]. Primary outcomes included the presence of multiple fractures (≥ 2 acute OVFs) and length of hospital stay. Secondary outcomes included time to postoperative mobilization (defined as day to standing/walking with/without aid), and incidence of postoperative wound infections.

### Imaging and muscle quantification

**Fig. 2:**
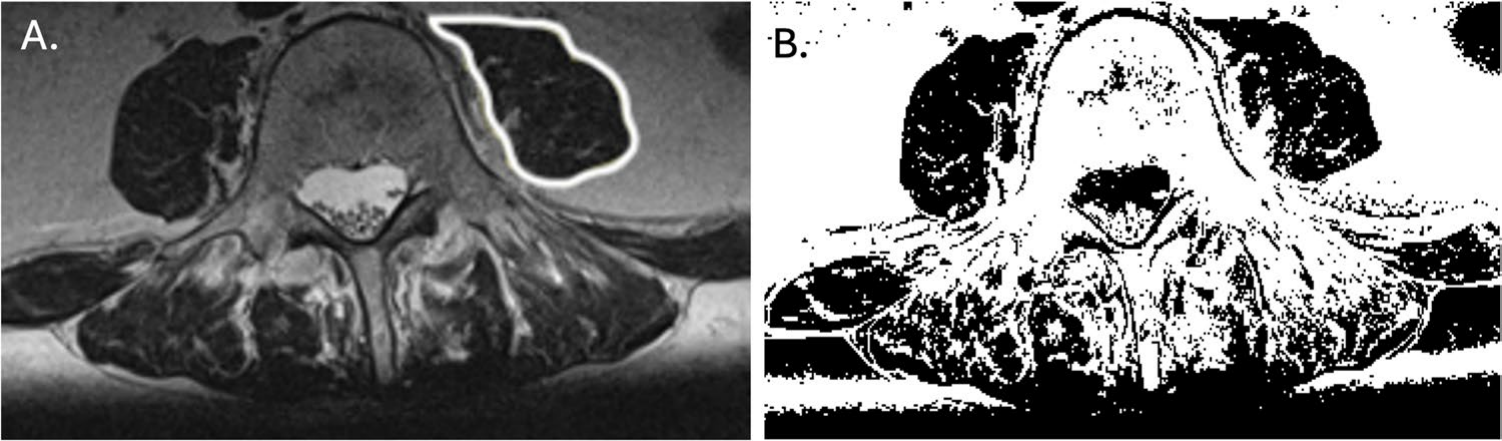
Schematic representation of MRI scans at L4 level **A**. Axial MRI scan demonstrating manual segmentation of the cross sectional area of the psoas muscle(pCSA). **B**. Axial MRI scan after signal intensity has been adjusted to identify fat infiltration indicated in white

Quantitative assessment of skeletal muscle was performed using axial T2-weighted MRI images at the L4 vertebral level. The cross-sectional area of psoas muscles (pCSA) was measured manually using ImageJ® image analysis software (version 1.54 g, National Institutes of Health, Bethesda, MD) and averaged after measuring the cross-sectional area bilaterally. Two indices were calculated for normalization: the psoas muscle index (PMI = psoas CSA [cm^2^]/height^2^ [m^2^]) and the psoas to lumbar vertebral body area ratio (PLVI = psoas CSA [cm^2^]/vertebral body area [cm^2^]). Fatty infiltration of the psoas muscle was evaluated semi-quantitatively using ImageJ based on signal intensity characteristics as previously described [10]. Thresholds were manually set for each image, and the amount of fat infiltration (FI) was expressed in % (FI = (FAT/pCSA) × 100), and the functional cross-sectional area (fCSA) was determined as the difference (pCSA − FAT) (see Fig. 2). Sarcopenia was defined according to established PMI cutoff values as lower bound (mean − 2 SD) as described previously [11, 12].

### Statistical analysis

Data were presented as mean ± standard deviation (SD) for normally distributed variables or as median with interquartile range (IQR) for non-normally distributed data. Normality of distribution was assessed using the Shapiro–Wilk test. Continuous variables were compared between groups using the independent sample *t*-test or the Mann–Whitney *U* test. Categorical variables were compared using the chi-square test or Fisher’s exact test, as appropriate based on expected frequencies. Correlation between variables was performed using either Spearman or Pearson correlation as appropriate. To investigate the association between sarcopenia and the presence of multiple vertebral fractures, logistic regression analysis was performed with adjustment for potential confounders including age, sex, and serum albumin levels. Results were reported as odds ratios (ORs) with corresponding 95% confidence intervals (CIs). Correction for multiple comparisons was done using Bonferroni when appropriate. A two-tailed *p*-value < 0.05 was considered indicative of statistical significance. All analyses were conducted using SPSS® software (IBM Corp. 2021. IBM SPSS Statistics for Macintosh, Version 28.0. Armonk, NY: IBM Corp).

## Results

### Demographics

A total of 142 patients were included, with a median age of 81 years (IQR 74–85). The cohort comprised 94 female (66.2%) and 48 male patients (33.8%). Sarcopenia was identified in 103 patients (72.5%) using pre-defined PMI cutoff values. Patients with sarcopenia were older and had a significantly lower BMI as compared to those without sarcopenia (see Table 1).

**Table 1:** Demographics and clinical presentation

	Sarcopenia (n=103)	Non-sarcopenia (n=39)	*p*-value
Age (years)	81 (75 – 85)	77 (69 – 86)	0.181
BMI (kg/m^2^)	24.5 (22.1 – 27.4)	26.9 (24.2 – 31.2)	**0.004**
ASA	2.6 ± 0.7	2.7 ± 0.7	0.429
Laboratory parameters			
Albumin (g/l)	34.7 ± 4.7	39.4 ± 4.8	**0.021**
Hb (g/dl)	11.2 ± 1.3	13.2 ± 1.6	**0.017**
Creatinine (mmol/l)	80.3 ± 28.9	89.1 ±16.6	0.735
BUN (mmol/l)	17.8 ± 57.7	5.4 ± 1.6	0.304
Total number of fractures	181 (79.4%)	47 (20.6%)	
No. of Vertebral fractures per patient, *n (%)*			**0.018**
Single	64 (62.1)	32 (82.1)	
Multiple*	39 (37.9)	7 (17.9)	
OF classification, n(%)			0.345
OF 1	2 (1.1%)	0	
OF 2	49 (27.1%)	12 (25.5%)	
OF 3	60 (33.1%)	19 (40.4%)	
OF 4	64 (35.4%)	15 (31.9%)	
OF 5	6 (3.3%)	1 (2.1%)	
Osteoporosis Parameters			
DXA-Scan			
T-score Lumbar spine	-1.8 ± 1.5	-0.9 ± 1.8	**0.026**
T-score Femoral neck	-1.9 ± 1	-1.8 ± 1.1	0.59
T-score Hips	-1.7 ± 1.2	-1.6 ± 1.2	0.79
HU in CT	70.4 ± 24.7	78.9 ± 26.8	0.145
Sarcopenia Parameters			
pCSA (cm^2^)	16.3 ± 3.3	18.7 ± 4.5	**<0.001**
pMI (cm^2^/m^2^)	5.9 ± 1.1	6.6 ± 1.3	**0.001**
pLVI (cm^2^/cm^2^)	1.2 ± 0.3	1.3 ± 0.3	0.079
FI (%)	23 ± 8.9	17.4 ± 6.9	**<0.001**
fCSA (cm^2^)	6.2 ± 2	10.6 ± 2.9	**<0.001**

*ASA* – American Society of Anesthesiologists, *BMI* – Body Mass Index, *BUN* – Blood Urea Nitrogen, *NRS* – Numerical Rating Scale, *HU* – Hounsfield units, *pCSA* – Psoas Cross Sectional Area, *pMI* – Psoas Muscle Index, *pLVI* - Psoas to Lumbar Vertebral body area ratio, *FI* – Fat Infiltration, *fCSA* – Functional Cross Sectional Area. Bolded entries indicate significant differences.

*Multiple – defined as two or more concurrent vertebral fractures at presentation

### Clinical parameters

Patients with sarcopenia presented with a lower serum hemoglobin and albumin values in serum but showed no difference in creatinine or preoperative risk profile measured by the ASA (American Society of Anesthesiologists) score. Pain at presentation as measured by the NRS (numerical rating scale) was similar in both groups (see Table 2).

**Table 2:** Sarcopenia and clinical outcome

	Sarcopenia (n=103)	Non-sarcopenia (n=39)	*p*-value
Therapy, *n(%)*			0.825
Surgical	81 (78.6)	30 (76.9)	
Conservative	22 (21.4)	9 (23.1)	
Postoperative length of stay, days*	5.6 ± 5.5	7.7 ± 17.8	0.458
Total in-hospital stay, days	14.9 ± 11.8	14.8 ± 18	0.953
Days to standing, days	1.7 ± 1.9	1.3 ± 1.4	0.262
Days to walking, days	2.2 ± 2.6	1.9 ± 2.3	0.626
Postoperative wound infection, *n(%)*	4 (3.9)	4 (10.3)	0.142
Pain (NRS Scale)			
At admission	8 (8 – 10)	8 (8 – 9.8)	0.341
Postoperatively	5 (4 – 7)	6 (4 – 7)	0.804
Upon discharge	4 (3 – 5)	4 (3 – 5)	0.701

*NRS* – Numeric rating scale

*Analysis only for surgically treated patients

### Vertebral fractures

**Fig. 3:**
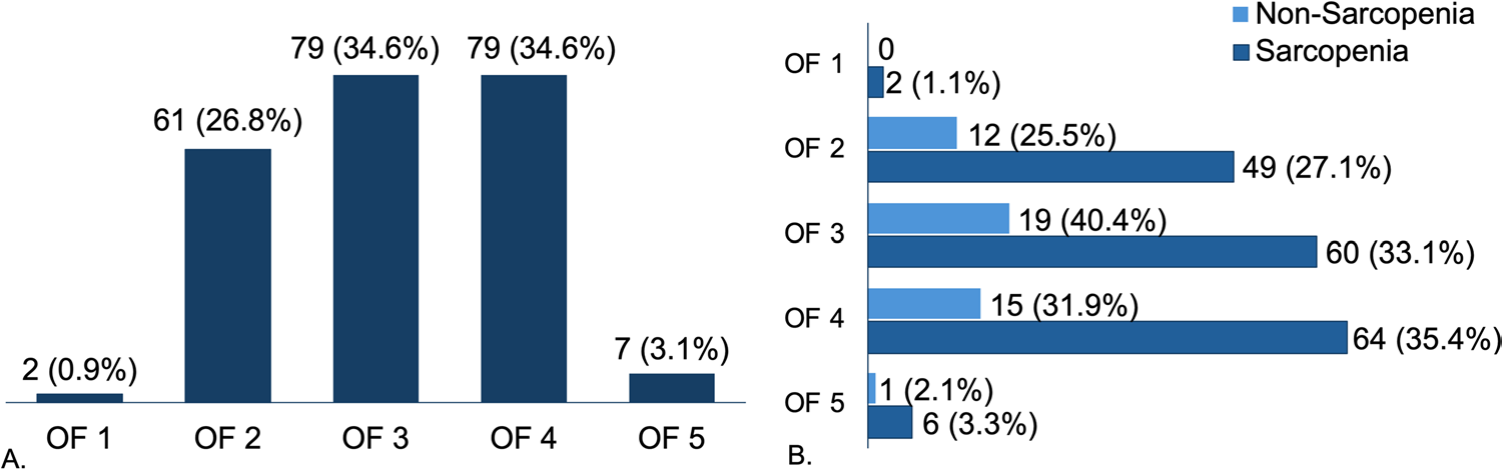
OF classification of osteoporotic vertebral fractures **A**. OVFs diagnosed in the entire cohort categorized according to the OF classification, **B**. Distribution of OVFs according to the OF classifications in the sarcopenia and non-sarcopenia group

A total of 228 fractures in 142 patients were diagnosed in this cohort. Using the OF classification [13], the most commonly seen fracture types were OF 3 (34.6%) and OF 4 (34.6%). In total, 26.8% were OF 2 and 3.1% were OF 5 (see Fig. 3). A total of 181 fractures were seen in patients with sarcopenia and 47 in the non-sarcopenia group. A significantly higher number of patients in the sarcopenia group (37.9% vs 17.9%) presented with multiple acute vertebral fractures, defined as two or more vertebral fractures. With regard to OF classification, OF 4 predominated (35.4%) in the sarcopenia group whereas OF 3 (40.4%) predominated in the non-sarcopenia group. A logistic regression model showed that patients in the sarcopenia group were 2.78 (95% CI; 1.1–6.9, *p* = 0.027) times more likely to present with multiple vertebral fractures.

### Osteoporosis and Sarcopenia Parameters

**Fig. 4:**
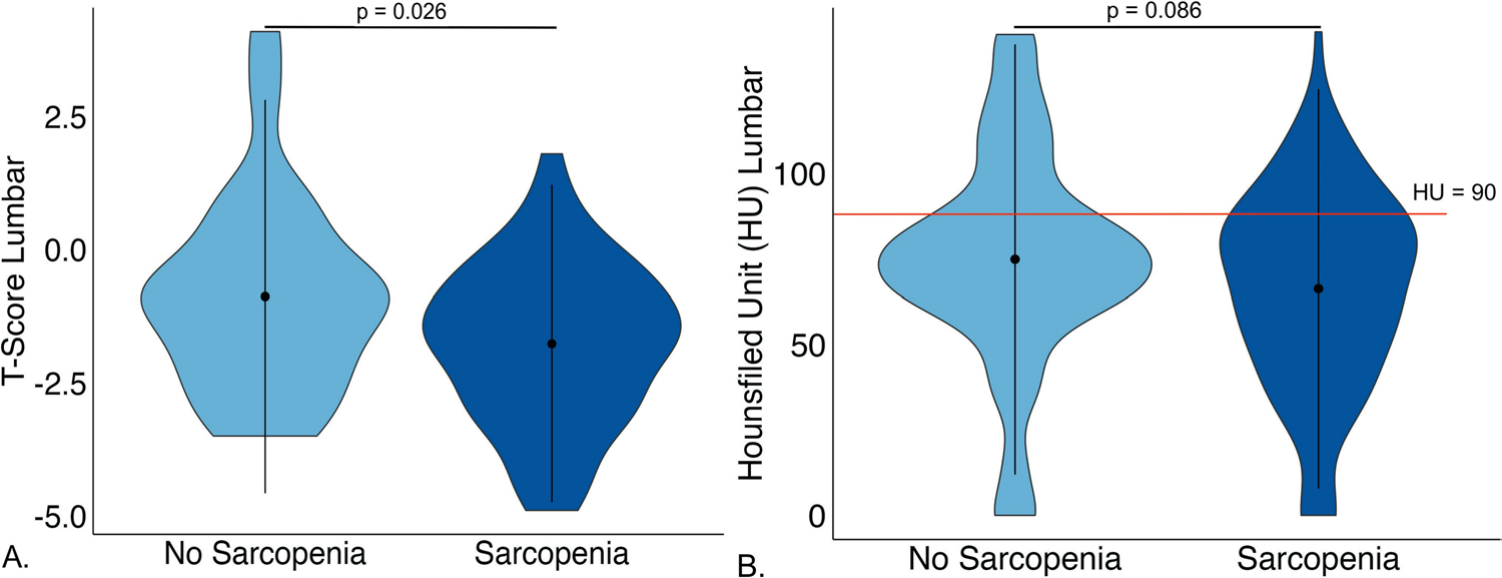
Differences in osteoporotic parameters between groups A.Significant difference in T-score lumbar spine between sarcopenia and non-sarcopenia. B. No significant difference in HU-measurement in CT between the two groups. The redline indicates the cut-off value for osteoporosis

**Fig. 5:**
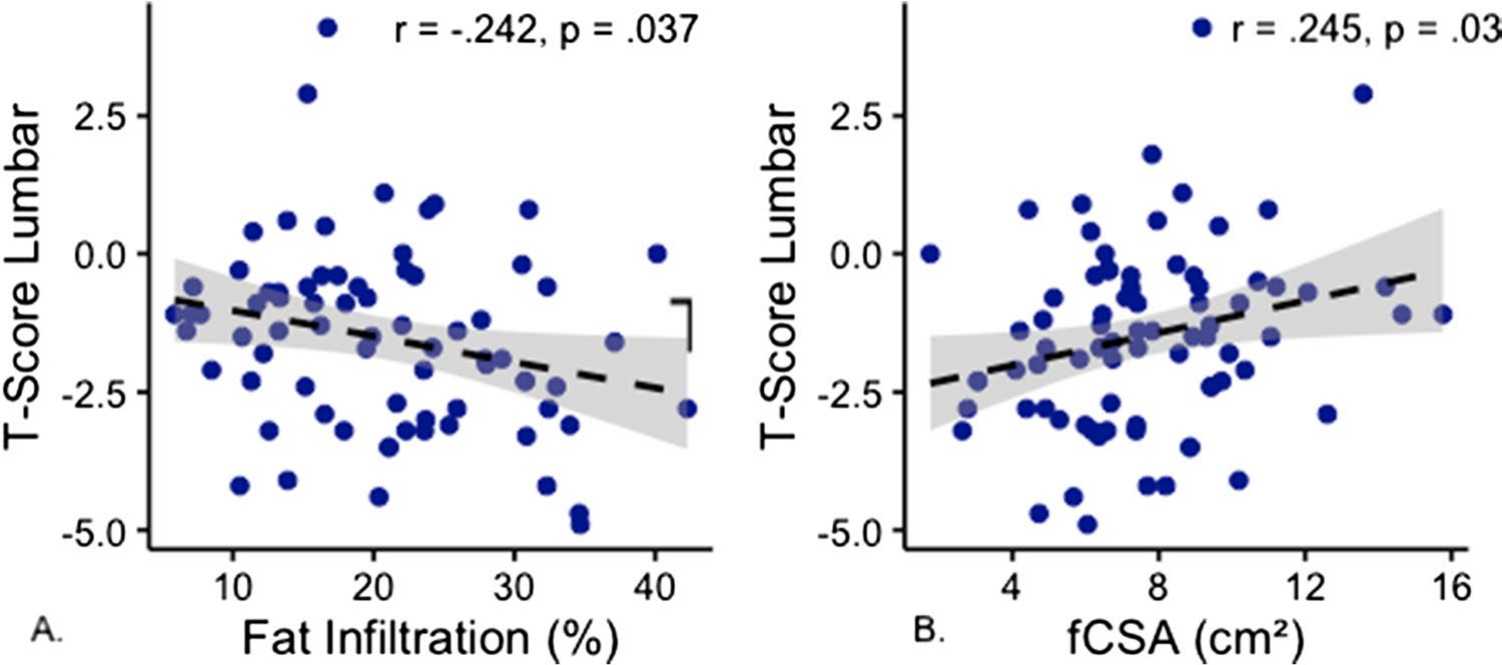
Correlation of fat infiltration (FI) and functional cross-sectional area (fCSA) with T-scores of the lumbar spine. A. a negative correlation between FI and T-score indicating that higher FI is associated with lower T-score, B. a positive correlation between fCSA and T-score indicating that higher fCSA is associated with higher T-scores.

*T*-scores of the lumbar spine scans were determined. Patients in the sarcopenia group had a significantly lower mean *T*-score than patients in the non-sarcopenia group (−1.8 ± 1.5 vs −0.88 ± 1.8, *p* = 0.026; see Fig. 4). No statistically significant differences were seen in *T*-scores of femoral neck and hip between the two groups. CT HU measurements indicated a total of 106 (74.6%) of the entire cohort to be in the osteoporotic range (HU < 90 [14]). Among these, 28 (26.4%) were in the non-sarcopenia group vs 78 (73.6%) were in the sarcopenia group. No statistical difference was seen between the two groups with regard to HU (*p* = 0.404). Patients in the sarcopenia group presented with a significantly smaller pCSA, higher FI, and lower fCSA (see Table 1). A significant negative correlation between FI and lumbar *T*-score (*r* = −0.242, *p* = 0.037) and positive correlation between fCSA and lumbar *T*-score (*r* = 0.245, *p* = 0.030) was seen (see Fig. 5). Patients with multiple osteoporotic vertebral fractures had higher FI (22.9 ± 8.8. vs 20.8 ± 8.7, *p* = 0.102) and lower fCSA (7 ± 3.1 vs 7.6 ± 2.9, *p* = 0.137). However, these results were not statistically significant.

### Association of sarcopenia and clinical outcome

There was no statistically significant difference in clinical outcome parameters including pain postoperatively, upon discharge, choice of therapy, days to mobilization, length of hospital stay (postoperative and total in-hospital stay), and postoperative wound infection rates.

## Discussion

Our study investigated the prevalence of sarcopenia among patients with OVFs and its association with clinical parameters and outcomes. We found a high prevalence of sarcopenia (72.5%) in our cohort. Sarcopenia patients demonstrated lower *T*-scores, lower BMI, and serum albumin levels. Sarcopenia patients had a 2.78-fold higher risk of multiple OVFs compared to non-sarcopenic patients. Furthermore, significant correlations between higher fat infiltration of the psoas muscle and *T*-score lumbar spine were seen, highlighting the relationship between osteoporosis and sarcopenia.

Sarcopenia and osteoporosis are closely related disorders more prevalent in older adults [8]. The prevalence of sarcopenia in osteoporotic post-menopausal women has been reported to be as high as 50% [15], while other studies reported a rate of 15.1% in women and 10.4% in men over the age of 80 [16]. Furthermore, the risk of coexisting osteoporosis in sarcopenia patients has been reported to be as high as fourfold [17]. The prevalence of sarcopenia in our cohort of patients with OVFs was 72.5%. The higher prevalence rate can be attributed to different factors, including specific parameters and cutoff values for the diagnosis of sarcopenia, and anthropometric variables of the study population, including age, sex, BMI, ethnic, and environmental factors [17–19]. Our findings also advocate for the integration of routine reporting of psoas muscle parameters in lumbar MRI scans for opportunistic sarcopenia screening. Similar to CT-based opportunistic osteoporosis screening, such MRI-based assessments may allow early identification of musculoskeletal frailty without additional imaging or radiation exposure [20, 21].

Our study further demonstrated the strong relationship between osteoporosis and sarcopenia evidenced by the significant correlations between sarcopenia parameters and the corresponding *T*-scores. This is in line with previous studies that have demonstrated the bidirectional relationship between the two pathologies. This relationship exists across multiple scales, including biological, mechanical, and lifestyle mechanisms. The “muscle-bone unit” hypothesis proposes that muscle deterioration leads to decreased mechanical load bearing of bones and disrupts muscle-bone crosstalk, reducing bone remodelling and maintenance [22, 23]. Additionally, systemic factors such as altered hormonal signaling and chronic inflammation contribute to musculoskeletal frailty and increased fracture risk [24]. Our observed coupling of muscle and bone metrics suggests that muscle fat infiltration might be a common pathological mediator of fragility. This also aligns with previous studies that have reported similar results even after adjustments for age, sex, and BMI [25–27].

Furthermore, our study reported that sarcopenia was associated with a significantly higher risk (2.78-fold) of multiple acute OVFs, further highlighting the clinical impact of sarcopenia. These results align with current evidence that demonstrated nearly a threefold higher odds of vertebral re-fracture compared to non-sarcopenic patients in a cohort of more than 2000 osteoporotic fracture patients [28]. Interestingly, non-sarcopenic patients were more likely to sustain only a single vertebral fracture. This may reflect the greater mechanical stability and energy absorption provided by preserved psoas muscle architecture, which helps attenuate axial load transfer and reduce sequential vertebral failure [29, 30]. In contrast, sarcopenic individuals exhibit reduced muscle mass and fatty degeneration, which impair spinal support, decrease dynamic stabilization, and increase susceptibility to multiple fractures under similar biomechanical stress conditions. These findings reinforce the role of muscle integrity as a critical determinant of spinal resilience in osteoporotic patients.

Despite the above associations, we did not observe significant differences in clinical outcomes, including mobilization and surgical site infections. Both groups had comparable pain reduction after treatment and similar times to mobilization. This can be due to individually tailored pain management despite standard care, early mobilization, and surgical stabilization in the very acute phases. Our study’s follow-up was limited to discharge; subtle differences in functional recovery might manifest later. Recent literature has shown that sarcopenic patients were more likely to experience persistent pain during recovery. Li et al. found that in patients undergoing kyphoplasty for OVFs, those with sarcopenia had a twofold higher incidence of residual back pain at 1month postoperatively [31]. Yin et al. focusing on older adults with osteoporotic thoracolumbar fractures treated with kyphoplasty reported that sarcopenic patients had longer rehabilitation times and significantly higher 3-year mortality than non-sarcopenic patients [32].

Our study has limitations. This study was performed retrospectively in a relatively small sample size comprising of Caucasian patients in a single center.In addition, only patients with MRI were included, with a potential for selection bias. This limits generalization to a broader population. Furthermore, predefined PMI cutoff values were used. The determination of population-specific cutoff values and the inclusion of other sarcopenia assessment methods, including muscle strength and physical performance, could have increased the reliability of our findings. In addition, clinical outcomes were only assessed before discharge. Long-term follow-up can assist in highlighting the clinical importance of sarcopenia in postoperative recovery and quality of life in these patients.

## Conclusion

In conclusion, our study demonstrates the significant association between sarcopenia and increased fracture risk in patients with OVFs. The clinical implications of our findings underscore the importance of prevention, early recognition and management of sarcopenia in this frail population. Interventions aimed at preserving or improving muscle mass and function may mitigate fracture risk and improve overall outcomes in this vulnerable population. Future research should focus on prospective, multi-center studies to validate these findings across diverse patient demographics and clinical settings.

## Data Availability

All data supporting the findings of this study are included within the article and any further clarifications can be obtained from the corresponding author upon reasonable request.

## Abbreviations

*ASA*: American Society of Anesthesiologists
*BMD*: Bone mineral density
*BMI*: Body mass index
*BUN*: Blood urea nitrogen
*CI*: Confidence interval
*CSA*: Cross-sectional area
*CT*: Computed tomography
*DXA*: Dual-energy X-ray absorptiometry
*fCSA*: Functional cross-sectional area
*FI*: Fat infiltration
*HU*: Hounsfield units
*IQR*: Interquartile range
*MRI*: Magnetic resonance imaging
*NRS*: Numerical rating scale
*OF*: Osteoporotic fracture (classification system of the DGOU)
*OR*: Odds ratio
*OVFs*: Osteoporotic vertebral fracture(s)
*pCSA*: Psoas cross-sectional area
*PLVI*: Psoas-to-lumbar vertebral body index (pCSA/vertebral body area)
*PMI*: Psoas muscle index (pCSA/height^2^)
*SD*: Standard deviation STIR - Short-Tau-Inversion-Recovery
